# Supplementary material for: Empirical methods for controlling false positives and estimating confidence in ChIP-Seq peaks
Source: BMC Bioinformatics. 2008 Dec 5;9:523. doi: 10.1186/1471-2105-9-523 (PMC2628906; doi:10.1186/1471-2105-9-523)
Supplement: Additional File 1 — A variety of html documents from the USeq web site detailing the available applications, their best usage, output file type descriptions, command line menus, etc. [file 1471-2105-9-523-S1.zip › USeqUserGuides/JavaDoc.zip!/JavaDoc/trans/misc/CalcGCRatiosFromIntervals.html]

CalcGCRatiosFromIntervals


|  |  |  |
| --- | --- | --- |
| **Overview** \| **Package** \| **Class** \| **Use** \| **Tree** \| **Deprecated** \| **Index** \| **Help** | |  |
| **PREV CLASS**   **NEXT CLASS** | **FRAMES**    **NO FRAMES**     **All Classes** |  |
| SUMMARY: NESTED \| FIELD \| CONSTR \| METHOD | DETAIL: FIELD \| CONSTR \| METHOD |  |


---


## trans.misc Class CalcGCRatiosFromIntervals

```
java.lang.Object
  trans.misc.CalcGCRatiosFromIntervals
```

---

``` public class CalcGCRatiosFromIntervals extends java.lang.Object ```

Parse out treatment and control oligo info from intervals, calculate GC and ratios to look for bias.

---

**Constructor Summary**

`CalcGCRatiosFromIntervals()`


| **Method Summary** | |
| --- | --- |
| `static void` | `main(java.lang.String[] args)` |
| `static void` | `printCalculations(java.util.ArrayList doubleAL, int gc)`             Prints %GC, Mean, 3xStdErr |

**Methods inherited from class java.lang.Object**

`equals, getClass, hashCode, notify, notifyAll, toString, wait, wait, wait`

**Constructor Detail**

### CalcGCRatiosFromIntervals

```
public CalcGCRatiosFromIntervals()
```


**Method Detail**

### main

```
public static void main(java.lang.String[] args)
```

---


### printCalculations

```
public static void printCalculations(java.util.ArrayList doubleAL,
                                     int gc)
```

:   Prints %GC, Mean, 3xStdErr


---


|  |  |  |
| --- | --- | --- |
| **Overview** \| **Package** \| **Class** \| **Use** \| **Tree** \| **Deprecated** \| **Index** \| **Help** | |  |
| **PREV CLASS**   **NEXT CLASS** | **FRAMES**    **NO FRAMES**     **All Classes** |  |
| SUMMARY: NESTED \| FIELD \| CONSTR \| METHOD | DETAIL: FIELD \| CONSTR \| METHOD |  |


---
